# Supplementary material for: Social Determinants of Human Papillomavirus Vaccine Uptake Among Adolescent Girls in Low-Middle-Income Countries: A Systematic Review & Meta-Analysis
Source: Inquiry. 2025 Dec 23;62:00469580251399368. doi: 10.1177/00469580251399368 (PMC12743787; doi:10.1177/00469580251399368)
Supplement: sj-docx-5-inq-10.1177_00469580251399368 – Supplemental material for Social Determinants of Human Papillomavirus Vaccine Uptake Among Adolescent Girls in Low-Middle-Income Countries: A Systematic Review & Meta-Analysis [file sj-docx-5-inq-10.1177_00469580251399368.docx]

**Supplementary File 2:** Database Search Strategy for Studies on Social Determinants of HPV Vaccine Uptake Among Adolescent Girls in Low-Middle-Income Countries (LMICs)

| Database | | Searching terms | Number of studies | Time period |
| --- | --- | --- | --- | --- |
| PubMed | ((hpv vaccine[Title/Abstract]) OR (human papillomavirus vaccine[Title/Abstract])) AND ((uptake[Title/Abstract]) OR (coverage[Title/Abstract]) AND (factors OR barriers OR determinants) AND (lmic[Title/Abstract] OR low middle income countries[Title/Abstract] OR low-middle-income countries[Title/Abstract]) | | 271 | January 2010 to February 2025 |
| Google scholar | “hpv vaccine” OR “human papillomavirus vaccine” AND “uptake” OR “coverage” AND “factors” OR “barriers” OR “determinants” AND “lmic” OR low middle income countries” | | 354 |  |
| Science Direct | “hpv vaccine” OR “human papillomavirus vaccine” AND “uptake” OR “coverage” AND “factors” OR “barriers” OR “determinants” AND “lmic” OR low middle income countries” | | 427 |  |
