# Supplementary material for: Social Determinants of Human Papillomavirus Vaccine Uptake Among Adolescent Girls in Low-Middle-Income Countries: A Systematic Review & Meta-Analysis
Source: Inquiry. 2025 Dec 23;62:00469580251399368. doi: 10.1177/00469580251399368 (PMC12743787; doi:10.1177/00469580251399368)
Supplement: sj-docx-6-inq-10.1177_00469580251399368 – Supplemental material for Social Determinants of Human Papillomavirus Vaccine Uptake Among Adolescent Girls in Low-Middle-Income Countries: A Systematic Review & Meta-Analysis [file sj-docx-6-inq-10.1177_00469580251399368.docx]

**Supplementary Table 1:** **Risk of Biasness in the current study according to the JBI checklist**

| **Author** | **Were the criteria for inclusion in the sample clearly defined?** | **Were the study subjects and the setting described in detail?** | **Was the exposure measured in a valid and reliable way?** | **Were objective, standard criteria used for measurement of the condition?** | **Were confounding factors identified?** | **Were strategies to deal with confounding factors stated?** | **Were the outcomes measured in a valid and reliable way?** | **Was appropriate statistical analysis used?** | **Weight (%age)** | **Overall critical appraisal** |
| --- | --- | --- | --- | --- | --- | --- | --- | --- | --- | --- |
| **Hailu et al^18^** | 1 | 1 | 0 | 1 | 1 | 1 | 0 | 1 | 75% | **≈** |
| **Nhumba et al^19^** | 1 | 1 | 1 | 1 | 1 | 1 | 1 | 1 | 100% | **✔** |
| **Josephat et al^20^** | 1 | 1 | 1 | 1 | 1 | 1 | 1 | 1 | 100% | **✔** |
| **Kassa et al^21^** | 1 | 1 | 0 | 1 | 0 | 1 | 0 | 1 | 62.5% | **≈** |
| **Aruho et al^22^** | 1 | 1 | 1 | 1 | 1 | 0 | 1 | 0 | 75% | **≈** |
| **Ndibazza et al^23^** | 1 | 1 | 1 | 0 | 1 | 1 | 1 | 1 | 87.5% | **✔** |
| **Isabriye et al^24^** | 1 | 1 | 1 | 1 | 1 | 0 | 1 | 1 | 87.5% | **✔** |
| **Nabriye et al^25^** | 1 | 1 | 1 | 1 | 1 | 1 | 1 | 1 | 100% | **✔** |

**1=** Yes (Y); 0**=** No (N)/ Unclear (U)/ Not applicable (NA); **✔**= Low-risk of bias (80–100%); **≈**= Moderate-risk of bias (50–80%); 🗙= High-risk of bias (20–50%)
