# Supplementary material for: Social Determinants of Human Papillomavirus Vaccine Uptake Among Adolescent Girls in Low-Middle-Income Countries: A Systematic Review & Meta-Analysis
Source: Inquiry. 2025 Dec 23;62:00469580251399368. doi: 10.1177/00469580251399368 (PMC12743787; doi:10.1177/00469580251399368)
Supplement: sj-docx-7-inq-10.1177_00469580251399368 – Supplemental material for Social Determinants of Human Papillomavirus Vaccine Uptake Among Adolescent Girls in Low-Middle-Income Countries: A Systematic Review & Meta-Analysis [file sj-docx-7-inq-10.1177_00469580251399368.docx]

**Supplementary Table 2.** Summary of determinants influencing HPV vaccine uptake in LMICs.

| **Study** | **Determinants** | | **AOR/APR****  **(95% CI)** |
| --- | --- | --- | --- |
| **Hailu et al**(34) | **Residence** | Rural | 1 (Ref.) |
|  |  | Urban | 3.46 (1.95-6.15)* |
|  | **Mother’s Education** | No formal education | 1 (Ref.) |
|  |  | Primary & High school | 1.20 (0.61-2.37) |
|  |  | College & above | 1.98 (0. 98-4.22) |
|  | **Father’s Educational status** | No formal education | 1 (Ref.) |
|  |  | Primary & High school | 1.20 (0.61-2.37) |
|  |  | College & above | 1.98 (0. 927-4.215) |
|  | **Mother’s Occupation** | Not Employed | 1 (Ref.) |
|  |  | Employed | 1.14 (0.453-2.74) |
|  | **Father’s Occupation** | Not Employed | 1 (Ref.) |
|  |  | Employed | 1.01 (0.56-1.83) |
|  | **Wealth index** | Poor | 1 (Ref.) |
|  |  | Middle | 0.585 (0.299-1.4) |
|  |  | Rich | 1.10 (0.43-2.79) |
|  | **Living status** | Sibling/ Relative | 1 (Ref.) |
|  |  | Single parent | 0.66 (0.28-1.58) |
|  |  | Both parents | 1.36 (0.68-2.73) |
|  | **Key decision-maker in the household** | Teachers/ peers | 1 (Ref.) |
|  |  | Family | 0.696 (0.19-2.43) |
|  |  | Myself | 1.64 (0.495-5.418) |
| **Kassa et al**(35) | **Residence** | Urban | 1 (Ref.) |
|  |  | Rural | 0.12 (0.07-0.21)* |
|  | **Religion** | Orthodox | 1 (Ref.) |
|  |  | Muslim | 1.97 (0.93-4.17) |
|  |  | Protestant | 1.54 (0.53-4.42) |
|  | **Father’s Educational status** | No formal education | 1 (Ref.) |
|  |  | Elementary | 0.85 (0.51-1.42) |
|  |  | Secondary | 0.77 (0.33-1.8) |
|  |  | College & above | 1.9 (0.71-4.9) |
|  | **Mother’s Occupation** | Farmer | 1 (Ref.) |
|  |  | Housewife | 1.54 (0.76-3.1) |
|  |  | Private | 0.5 (0.18-1.5) |
|  |  | Government | 0.45 (0.19-1.06) |
| **Nhumba et al**(36) | **Age (in years)** | 14+ | 1 (Ref.) |
|  |  | 12-13 | 0.14 (0.03-0.59)* |
|  | **Parental Educational level** | Secondary or above | 1 (Ref.) |
|  |  | No formal or Primary education | 0.61 (0.30-1.22)* |
|  | **Parent's Occupation** | Unemployed | 1 (Ref.) |
|  |  | Employed | 0.39 (0.14-1.05)* |
|  |  | Self Employed | 0.70 (0.28-1.73) |
|  | **Wealth index** | Highest | 1 (Ref.) |
|  |  | Lowest | 1.39 (0.57-3.38) |
|  |  | Second | 2.07 (0.87-4.88) |
|  |  | Middle | 1.52 (0.65-3.53) |
|  |  | Fourth | 0.57 (0.22-1.48) |
| **Josephat et al**(37) | **Religion** | Muslim | 1 (Ref.) |
|  |  | Christian | 1.19 (0.67-1.79)* |
|  | **Parental Educational level** | Informal education | 1 (Ref.) |
|  |  | Higher education | 1.96 (0.66-2.15)* |
|  |  | Secondary education | 1.61 (0.33-1.36)* |
|  |  | Primary education | 2.88 (0.71-5.19) |
|  | **Head of household** | Father/ Mother | 1 (Ref.) |
|  |  | Grandparents | 0.84 (0.58-2.44)* |
|  |  | Others | 4.75 (1.29-24.88) |
| **Aruho et al**(38) | **Age (in years)** | 16 | 1 (Ref.) |
|  |  | 15 | 0.920 (0.575-1.472) |
|  | **Religion** | Muslim | 1 (Ref.) |
|  |  | Christian | 1.015 (0.554-1.860) |
|  | **Parental Educational level** | Post-secondary | 1 (Ref.) |
|  |  | Primary school | 0.985 (0.871-1.115) |
|  |  | Secondary school | 0.989 (0.878-1.114) |
|  | **Adolescent stays with both parents** | No | 1 (Ref.) |
|  |  | Yes | 1.037 (0.955-1.126) |
|  | **Living status** | Friends | 1 (Ref.) |
|  |  | Mother only | 0.769 (0.595-0.995)* |
|  |  | Father only | 0.801 (0.582-1.103) |
|  |  | Relatives | 0.848 (0.612-1.174) |
|  | **Parent’s Marital status** | Separated | 1 (Ref.) |
|  |  | Married | 0.941 (0.872-1.014) |
|  |  | Single | 0.859 (0.776-0.951)* |
|  | **Key decision-maker in the household** | The adolescent herself | 1 (Ref.) |
|  |  | One of the parents | 0.984 (0.769-1.258) |
|  |  | Both parents | 1.071 (0.838-1.368) |
|  |  | The entire family | 1.023 (0.786-1.330) |
|  | **Relationship of decision maker/ household head with the adolescent** | Grandparent | 1 (Ref.) |
|  |  | Father | 1.050 (0.418-2.636) |
|  |  | Mother | 0.876 (0.385-1.992) |
|  |  | Guardian | 0.884 (0.277-2.818) |
|  |  | Auntie | 1.400 (0.238-8.250) |
| **Ndibazza et al**(39) | **Mother’s Education** | Uneducated/ No formal education | 1 (Ref.) |
|  |  | Primary | 0.65 (0.18-2.37) |
|  |  | Secondary | 0.66 (0.19-2.27) |
|  |  | Tertiary | 1.529 (0.43-5.34) |
|  |  | University | 1.24 (0.31-4.95) |
|  | **Mother’s/ Caretaker's Occupation** | Peasant | 1 (Ref.) |
|  |  | Business | 1.35 (0.73-2.47) |
|  |  | Hairdresser | 0.81 (0.15-4.31) |
|  |  | Civil servant | 1.78 (1.00-3.18)* |
|  |  | Housewife | 0.99 (0.34-2.93) |
|  | **Mother's Marital status** | Single | 1 (Ref.) |
|  |  | Married | 1.03 (0.579-1.79) |
|  |  | Divorced/ Separated | 0.72 (0.32-1.60) |
|  |  | Widowed | 1.21 (0.45-3.27) |
|  | **Head of household** | Father | 1 (Ref.) |
|  |  | Mother | 0.90 (0.54-1.46) |
|  |  | Guardian | 1.01 (0.48-2.15) |
|  |  | Brother/ sister | 0.37 (0.44-3.10) |
|  | **Number of people in household** | 1-5 | 1 (Ref.) |
|  |  | 6-10 | 0.80 (0.50-1.31) |
|  |  | 11-15 | 0.31 (0.10-0.89)* |
| **Isabriye et al**(40) | **Age (in years)** | 10 | 1 (Ref.) |
|  |  | 11 | 1.30 (1.06-1.61)* |
|  |  | 12 | 1.14 (0.94-1.39) |
|  |  | 13 | 1.40 (1.15-1.70)* |
|  |  | 14 | 1.41 (1.14-1.75)* |
|  | **Residence** | Rural | 1 (Ref.) |
|  |  | Urban | 0.92 (0.72-1.17) |
|  | **Number of people in household** | 1-8 | 1 (Ref.) |
|  |  | **≥ 9** | 0.81 (0.69-0.95)* |
|  | **Relationship to decision maker/ household head** | Daughter | 1 (Ref.) |
|  |  | Other | 0.94 (0.75-1.18) |
|  | **Wealth index** | Poorest | 1 (Ref.) |
|  |  | Poor | 1.11 (0.88-1.40) |
|  |  | Middle | 1.31 (1.01-1.69)* |
|  |  | Rich | 1.22 (0.93-1.59) |
| **Nabriye et al**(41) | **Age (in years)** | 9-10 | 1 (Ref.) |
|  |  | 11-12 | 1.3 (0.57-3.01)* |
|  |  | 13-15 | 1.2 (0.48-3.27) |
|  | **Religion** | Catholics | 1 (Ref.) |
|  |  | Anglicans | 1.1 (0.55-2.10) |
|  |  | Muslims | 0.5 (0.22-1.01) |
|  |  | Others | 0.9 (0.30-2.38) |
|  | **Education level of the caretaker** | None | 1 (Ref.) |
|  |  | Primary school | 0.7 (0.27-1.70) |
|  |  | Secondary & above | 1.6 (0.65-3.73) |
|  | **Mother's Occupation** | Housewife | 1 (Ref.) |
|  |  | Business woman | 5.9 (2.049-16.9)* |
|  |  |  |  |
|  |  | Formal Employment | 1.4 (0.49-3.72) |
|  |  | Farmer | 1.5 (0.579-3.95) |
|  | **Distance to Health Facility** | <1km | 1 (Ref.) |
|  |  | 1-3km | 1.9 (0.30-0.97) |
|  |  | >3km | 1.8 (0.21-1.6) |

***p-value**= ≤0.05

****AOR=** Adjusted Odds Ratio**; APR=** Adjusted Prevalence Ratio. **(*Note: The studies highlighted in blue reported data as APR, rest of the studies have reported data as AOR)***
