# Supplementary figures and images for: Social Determinants of Human Papillomavirus Vaccine Uptake Among Adolescent Girls in Low-Middle-Income Countries: A Systematic Review & Meta-Analysis
Source: Inquiry. 2025 Dec 23;62:00469580251399368. doi: 10.1177/00469580251399368 (PMC12743787; doi:10.1177/00469580251399368)

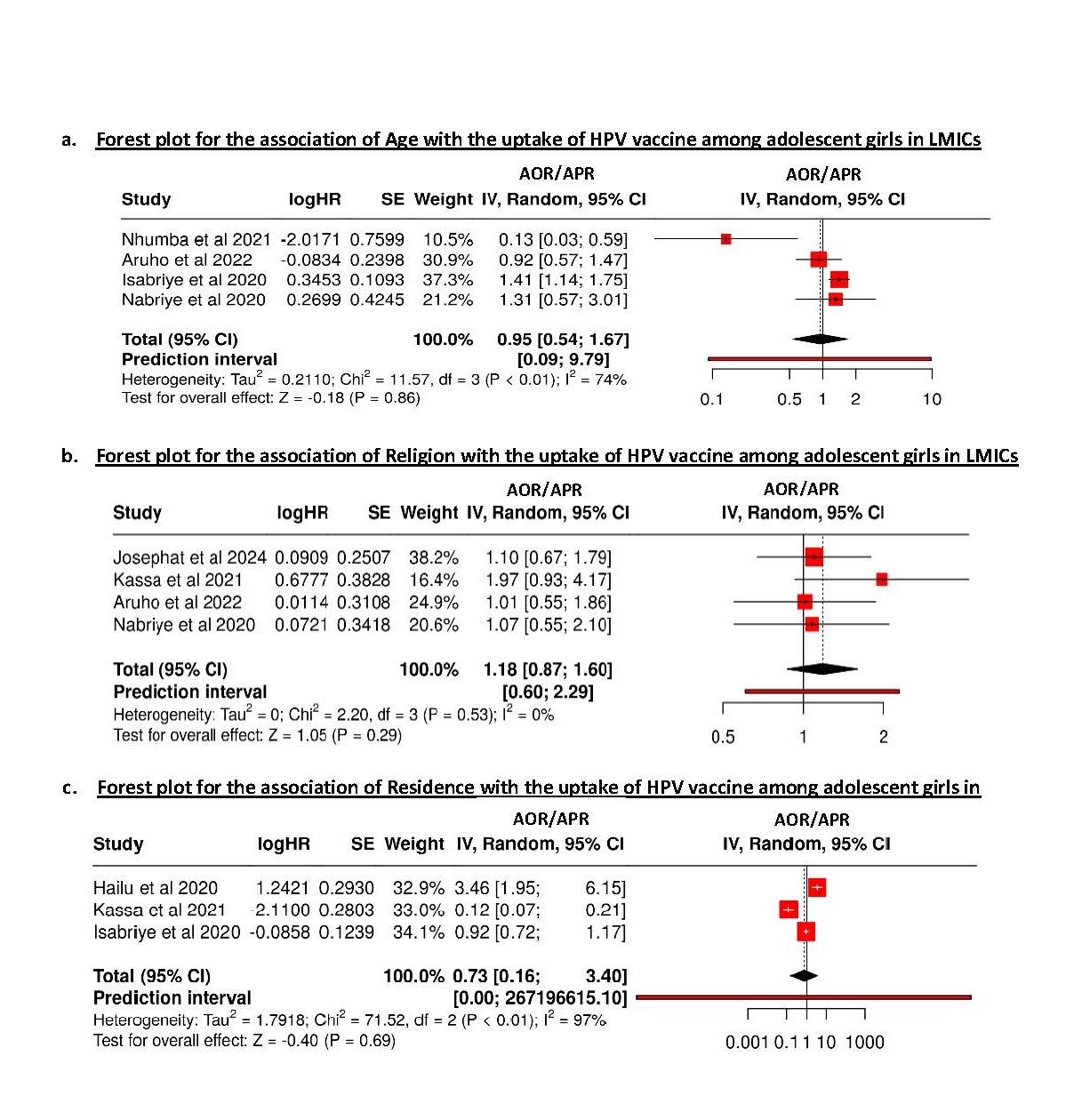

Supplement: sj-jpg-1-inq-10.1177_00469580251399368 – Supplemental material for Social Determinants of Human Papillomavirus Vaccine Uptake Among Adolescent Girls in Low-Middle-Income Countries: A Systematic Review & Meta-Analysis [file sj-jpg-1-inq-10.1177_00469580251399368.jpg]

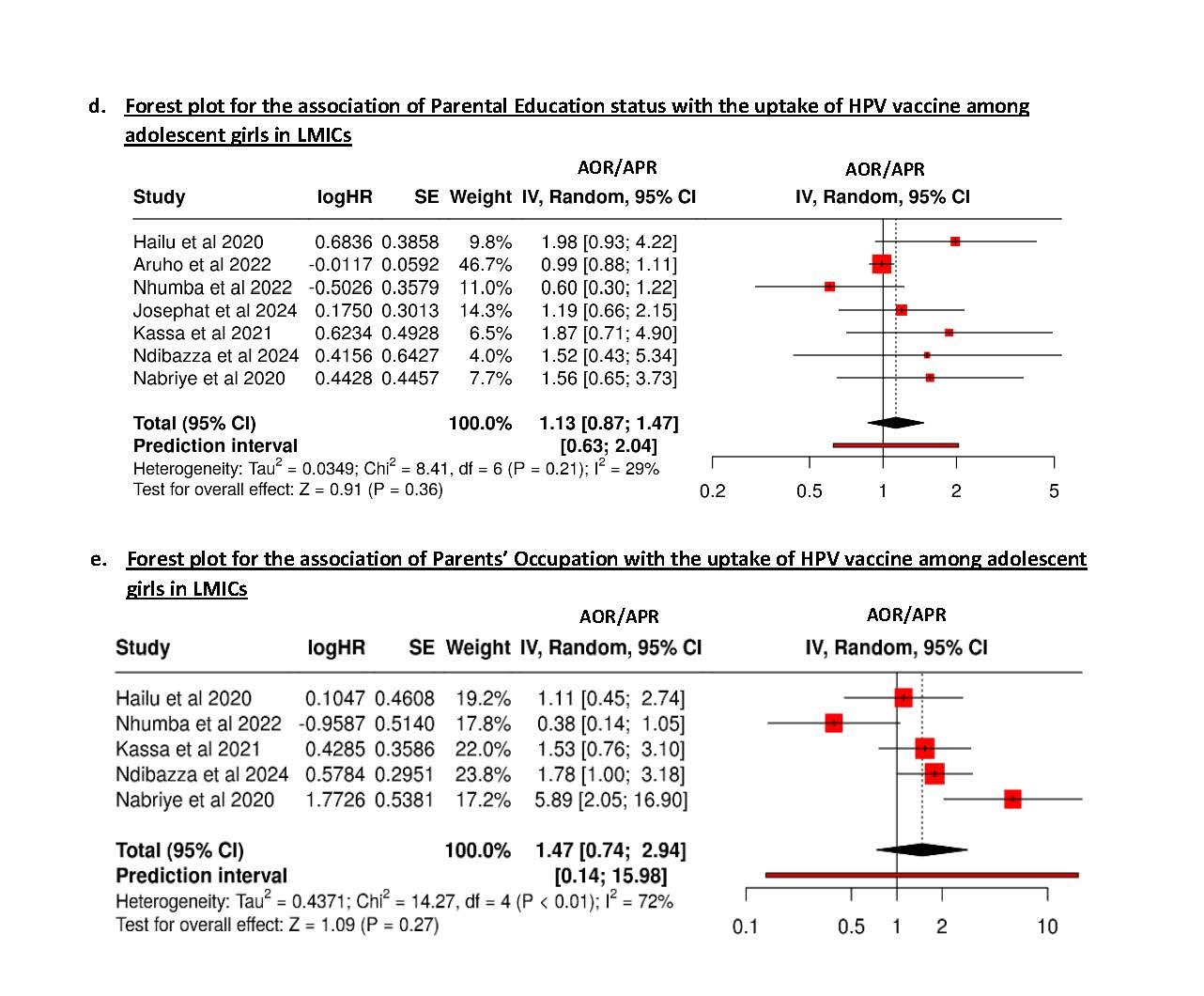

Supplement: sj-jpg-2-inq-10.1177_00469580251399368 – Supplemental material for Social Determinants of Human Papillomavirus Vaccine Uptake Among Adolescent Girls in Low-Middle-Income Countries: A Systematic Review & Meta-Analysis [file sj-jpg-2-inq-10.1177_00469580251399368.jpg]

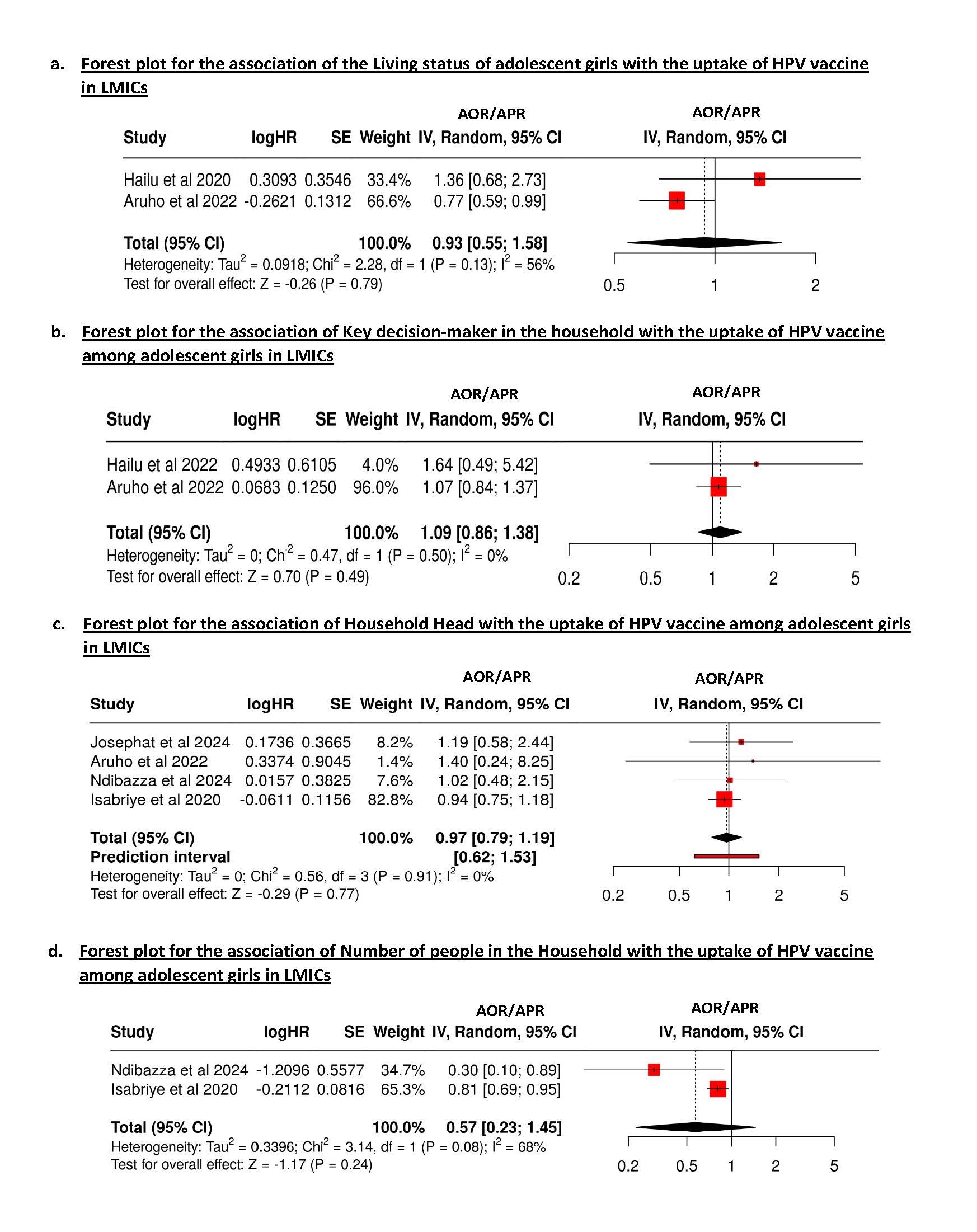

Supplement: sj-jpg-3-inq-10.1177_00469580251399368 – Supplemental material for Social Determinants of Human Papillomavirus Vaccine Uptake Among Adolescent Girls in Low-Middle-Income Countries: A Systematic Review & Meta-Analysis [file sj-jpg-3-inq-10.1177_00469580251399368.jpg]
